# Target Versus Modified-Standard Fortification of Human Milk and Growth of Very Low Birth Weight Infants: A Double-Blind Randomized Trial

**DOI:** 10.3390/nu18193241

**Published:** 2026-10-01

**Authors:** Corinna M. Gebauer, Rudolf G. Ascherl, Nadine Stamm, Julia Wiegand, Marina Stopsack, Annett Blaeser, Niels Rochow, Christoph Fusch, Walter A. Mihatsch, Ulrich H. Thome

**Affiliations:** 1 Division of Neonatology, Department of Pediatrics, University of Leipzig Medical Center, Liebigstraße 20a, 04103 Leipzig, Germany; 2 Institute of Clinical Chemistry and Laboratory Medicine, University Hospital Carl Gustav Carus, 01307 Dresden, Germany; 3 Department of Pediatrics, Paracelsus Medical University, 90471 Nuremberg, Germany; 4 Department of Pediatrics, University Medicine Rostock, 18057 Rostock, Germany; 5 Department of Pediatrics, McMaster University, Hamilton, ON L8S 4K1, Canada; 6 Department of Pediatrics, Ulm University, 89075 Ulm, Germany

**Keywords:** preterm infant, very low birth weight, human-milk fortification, target fortification, protein intake, growth, neurodevelopment, randomized controlled trial

## Abstract

**Background/Objectives:** Individualized (“target”) fortification of human milk is promoted to improve growth and subsequent neurodevelopment in very low birth weight (VLBW) infants, because standard fortifiers may not provide sufficient protein given the considerable intra- and interindividual variability of human-milk composition. We compared modified-standard fortification (MSF), an optimized high-protein standard regimen, with high-protein target fortification (TFO). **Methods:** In a single-center, double-blind randomized trial, VLBW infants (24 + 0 – 31 + 6 weeks) receiving 100 mL/kg/d of standard fortified human milk were randomized to MSF (protein and medium-chain triglycerides added to standard fortifier) or TFO until 36 weeks postmenstrual age (PMA). The primary outcome was weight-gain velocity from randomization to 36 weeks PMA; the trial was powered to detect a difference of 0.666 standard deviations. **Results:** Weight-gain velocity did not differ between the groups (MSF *n* = 54, TFO *n* = 52; MSF − TFO − 0.18 g/kg/d, 95% CI −1.02 to +0.67, *p* = 0.68), nor did anthropometry, z-scores, skinfold thickness or cranial widths at 36 weeks PMA, or Bayley-III scores at 24 months. **Conclusions:** Compared with optimized high-protein standard fortification, high-protein target fortification did not improve growth or neurodevelopmental outcomes.

## 1. Introduction

Very low birth weight (VLBW) infants need more protein and energy than what is provided by unfortified human milk. Multi-nutrient fortification is standard practice, although it increases in-hospital growth only modestly and has not been shown to improve later outcomes [1]. Still, VLBW infants are at high risk of postnatal growth failure due to limited nutrient reserves, physiological immaturity, and frequent co-morbidities. Adequate provision of macro- and micronutrients and especially protein during the early postnatal period is essential to support somatic growth and neurodevelopment [2,3]. The 2022 ESPGHAN position paper on enteral nutrition stresses that protein must be judged together with energy supply [4].

Standard human-milk fortification is based on estimated average milk composition and frequently falls short, because of the high intra- and interindividual variability of human-milk composition [5,6,7]. In addition, human-milk protein concentration decreases with postnatal age [8]. Individualized target fortification measures the composition of the milk actually fed and tailors supplementation to consensus targets [9]. A Cochrane review of individualized against standard fortification found moderate- to low-certainty evidence of short-term growth benefit [10], but the evidence base is small and no effect on neurodevelopment has been demonstrated [11].

Whether protein intakes beyond 3.5–4.0 g/kg/d improve growth and subsequent neurodevelopmental outcomes is unclear [3,12,13,14]. The most recent systematic review on the effect of enteral protein supplementation in preterm infants less than 32 weeks of gestation suggests protein intakes of 4.0–4.5 g/kg/d while stressing an appropriate protein-to-energy ratio [12]. An optimum and safe energy-to-protein ratio of 30 kcal per g of protein (equivalent to a protein-to-energy ratio of 3.3 g/100 kcal) has been proposed [15]. Since standard multicomponent human-milk fortifiers fall short of these goals, adding extra protein and energy to achieve high-protein intake and an energy-to-protein ratio of 30 kcal/g appears necessary.

The aim of the present double-blind randomized controlled trial was to compare the effect of high-protein target fortification (TFO) vs. modified-standard fortification (MSF), an optimized high-protein standard regimen in which a fixed protein supplement and medium-chain-triglyceride oil (MCT) were added to the human-milk feeds in addition to the multicomponent fortifier, on weight gain in VLBW infants.

## 2. Materials and Methods

### 2.1. Trial Design, Setting and Participants

This randomized, parallel-group, single-center, double-blind superiority trial (ISRCTN 13923832) was performed at the Division of Neonatology, Leipzig University Medical Center, Germany, a tertiary neonatal intensive care unit with one of the largest European milk banks. VLBW infants were enrolled from 21 June 2013 to 31 August 2016 until the planned sample size was reached, and included follow-up to 24 months corrected age. Registration was applied for with the ISRCTN registry on 29 January 2013, before the first infant was enrolled on 21 June 2013, and paid in October 2013; the registry completed the record only on 1 March 2018 and displays that date. Enrollment therefore began after registration was applied for, but before the record became public. No changes to eligibility criteria, interventions or outcomes were made after the trial commenced. No patients or members of the public were involved in the design, conduct or reporting.

Infants of 24 + 0–31 + 6 weeks gestation and 400–1499 g birth weight fed mother’s own milk, with parental consent to use donor milk, were eligible. Donor milk from the unit’s own milk bank was used in both groups when the mother’s supply was insufficient. Exclusion criteria were birth asphyxia, major congenital anomaly, abdominal surgery before randomization, clinically significant renal dysfunction, cholestasis (conjugated bilirubin > 60 µmol/L) and participation in another interventional trial. The institutional review board of the Medical Faculty of the University of Leipzig approved the study (082-13, 11 March 2013); written parental consent preceded inclusion.

### 2.2. Randomization and Blinding

As soon as the enteral feeding volume achieved 100 mL/kg/d of standard fortified human milk, the infants were stratified by sex and actual weight (<1000 g or ≥1000 g) and randomized in balanced blocks of four. Sequentially numbered, opaque envelopes containing the random group allocation were opened by the unit secretary, who was not involved in infant care. Parents, clinical staff and all outcome assessors were blinded to group allocation, and blinding of the investigator performing the Bayley-III assessment was maintained until follow-up at 24 months corrected age. Human milk was analyzed, fortified and handled by the milk bank team, which is operated by its own staff and not involved in direct patient care. Fortification according to allocation began with the next 24 h batch of feeds prepared after randomization, so no more than one day elapsed between allocation and the start of group-specific supplementation.

### 2.3. Feeding and Fortification

Progressive enteral feeding started on day 1. All infants received mother’s own milk or banked human milk. No infant formula was given. A feeding volume of 150 mL/kg/d was considered full enteral feeding. This upper volume limit could be exceeded at the discretion of nursing staff and attending physicians, e.g., when hunger or insufficient weight gain were anticipated.

Mother’s own milk was fed raw only from CMV-seronegative mothers, otherwise pasteurized (Holder: 62.5 °C, 30 min) until 32 weeks PMA; donor milk was left unpasteurized only if it came from CMV-seronegative donors. Pooled 24 h feeds were sonicated and analyzed daily by mid-infrared spectroscopy (Miris Human-Milk Analyzer, Uppsala, Sweden) [16]. Quality control followed the manufacturer’s protocol: the analyzer was cleaned after every tenth measurement, calibrated before the measurements of each day on which milk was analyzed, and validated internally once a year. A formal inter-assay validation was not performed. As an upper bound on analytical imprecision, we used the within-infant variation between consecutive measurement days, which also contains genuine biological change, expressed as the standard deviation of consecutive-day differences divided by √2 relative to the mean concentration. Mid-infrared analysis of fresh human milk has been shown to agree closely with chemical methods for protein and fat once device-specific calibration is applied [16]. The fortified milk itself was not re-analyzed: final macronutrient content was calculated from the measured native composition and the quantities of fortifier and supplements added.

In all infants fortification started with 2% multicomponent bovine-protein-derived fortifier (Aptamil Frauenmilchsupplement FMS^®^, Danone, Frankfurt am Main, Germany) once a feeding volume of 70 mL/kg/d was achieved, and increased over four days to 4.4%, which adds 1.1 g protein and 2.74 g carbohydrate per 100 mL. This 4.4% multicomponent fortification was identical in the two groups, and already fully established at the time of randomization, which occurred later when 100 mL/kg/d was achieved, and was continued unchanged in both arms for the whole intervention. The regimens differed only in the amount of modular protein and fat supplements added on top of it.

In the MSF group, human milk was supplemented in addition to the 4.4% multicomponent fortification with 1.0 g of protein powder (Aptamil Eiweiß+^®^, Danone, Frankfurt am Main, Germany), providing 0.82 g of protein, and 0.5 mL of medium-chain triglyceride oil (MCT; Ceres-Öl^®^, Dr. Schär, Burgstall, Italy), providing 0.45 g of fat, per 100 mL of fortified milk, throughout the intervention period. The MCT dose was chosen so that milk of average native fat content (3.9 g/100 mL, Section 3.2) would come close to the fat target of 4.5 g per 100 mL. Milk of average composition thus contained about 3.0 g of protein per 100 mL—1.05 g native protein, 1.10 g from the multicomponent fortifier (4.4%) and 0.82 g from the extra protein supplement. Neither dose was reduced as the infants matured, so MSF was over-fortified with protein after 34 + 0 weeks PMA, when the TFO target fell to 2.6 g per 100 mL.

In TFO each 24h feeding batch was supplemented based on its measured composition. Additional protein was added to achieve a target concentration of 3.0 g per 100 mL below 34 + 0 weeks PMA and 2.6 g per 100 mL thereafter, that is supplement (g/100 mL) = target − (measured native protein + 1.1 g/100 mL contributed by the fortifier), or zero when the sum already reached the target. MCT was added to achieve a fat concentration of 4.5 g per 100 mL, that is MCT (g/100 mL) = 4.5 − measured native fat, or zero when native fat already reached 4.5 g per 100 mL. No minimum or maximum dose was predefined for either supplement: both were prescribed per 100 mL of milk and infants were fed ad libitum, so the daily amount followed the volume taken. Energy was not itself a target and was not used to adjust either supplement. The concentration targets of 3.0 and 2.6 g per 100 mL were intended to provide intakes at the upper end of the ranges of 4.0–4.5 g/kg/d below 1000 g and 3.5–4.0 g/kg/d from 1000 to 1800 g [17] at a milk volume intake of 150–160 mL/kg/d.

The volume taken directly at the breast was quantified by test weighing, the infant being weighed immediately before and after each breastfeed on one of the ward’s electronic baby scales with a breast-milk-intake function (seca 354, seca, Hamburg, Germany; graduation 10 g) [18]. Milk taken at the breast was by definition unfortified and was assigned the measured macronutrient composition of that day’s analyzed pool. Daily macronutrient and energy intakes were computed from parenteral nutrition, the milk analysis and the fortifier added, relative to the milk actually consumed and the current weight.

### 2.4. Outcomes and Assessments

The primary outcome was weight gain from the start of the intervention to 36 + 0 weeks PMA; weight was measured every week on the baby scales used for test weighing (median interval 7 days; interquartile range 6–8), and length and head circumference about every two weeks (median interval 12 days; interquartile range 10–14). Weight-gain velocity was expressed by the exponential method of Patel and colleagues [19,20] and as the average accurate daily weight gain (AADWG) [19], taken as the median across the intervention of the daily weight increment divided by the mean of the two consecutive weights, in grams per kilogram per day. Nutritive efficiency was defined as AADWG divided by the mean daily enteral fluid intake per kilogram during the intervention period, expressed as grams per 100 mL. Secondary outcomes were anthropometry with z-scores at 36 + 0 weeks PMA (2013 Fenton reference via the University of Calgary application [21,22]; BMI by the Olsen LMS tables [23]), individualized growth trajectories [24], for which each infant’s weight was modeled forward from the postnatal weight minimum along its own percentile channel and the absolute difference between measured and modeled weight at 36 + 0 weeks PMA was compared between groups, and the change in weight z-score during the intervention. Further secondary outcomes were biceps skinfold thickness, interhemispheric and craniocortical widths on cranial ultrasound [25], and Bayley-III at 24 months corrected age by a blinded investigator [26]. Harms were assessed systematically by preprandial clinical chemistry every other week and plasma amino acids at 2, 4 and 7 weeks after randomization. Plasma amino acids are reported in the companion paper (Gebauer, Ascherl et al., submitted).

### 2.5. Statistical Analysis

The sample size calculation was based on weight gain (primary outcome). The expected absolute weight gain and its standard deviation (SD) were unknown. A sample size of 36 completed cases per group was required to detect a difference of 0.666 SD with 80% power and a two-sided α of 0.05, which was regarded to be clinically relevant. The planned sample size was increased to 55 infants per group to account for potential unequal variances between treatment groups and for post-randomization attrition. Unequal variability was anticipated because the individualized regimen was expected to affect not only mean nutrient intake but also the variability of achieved intakes. Attrition due to mortality and especially transfer to level 2 units before 36 weeks’ PMA is common in this population. No interim analyses were performed, and no stopping rules were prespecified. Final analysis was based on completed cases rather than the intention to treat because the primary outcome was expected to be unavailable in deceased or transferred infants.

Groups were compared by Welch two-sample t-test and chi-square test; data are mean ± SD, and confidence intervals derive from group summary statistics by the Welch approximation. Because the primary outcome was prospectively defined as a velocity over a fixed interval, the primary analysis used one summary value per infant. Because randomization was stratified, the primary outcome was additionally analyzed by linear regression adjusted for the two stratification variables entered as separate terms, sex and weight category (<1000 g or ≥1000 g) at randomization, which is the analysis consistent with the design [27]; the adjusted estimate is reported alongside the unadjusted comparison. As a further sensitivity analysis, all serial weights recorded during the intervention were modeled by a linear mixed-effects model with a random intercept and slope for each infant and a group-by-time interaction, fitted to the 526 weights actually measured during the intervention rather than to interpolated values, so that it uses the repeated measurements rather than one summary value per infant. Neither analysis was prespecified; both are reported as sensitivity analyses of the primary outcome.

Native milk composition was summarized with the infant as the unit of analysis: the mean of an infant’s daily measurements entered the between-group comparison. Variability is reported as the between-infant SD and coefficient of variation in these infant means and as the mean within-infant SD and coefficient of variation across days (Supplementary Table S1). We also report the proportion of study days on which the fixed MSF supplement, applied to the measured composition of that day’s pool, would have produced a protein concentration more than 0.2 g/100 mL away from the target concentration in force at that postmenstrual age.

No imputation and no multiplicity adjustment were applied. Analyses used R 4.0.3 [28] with plyr 1.8.6 [29], and nlme 3.1.168 for the mixed-effects model.

## 3. Results

### 3.1. Participants and Baseline Characteristics

**Figure 1:**
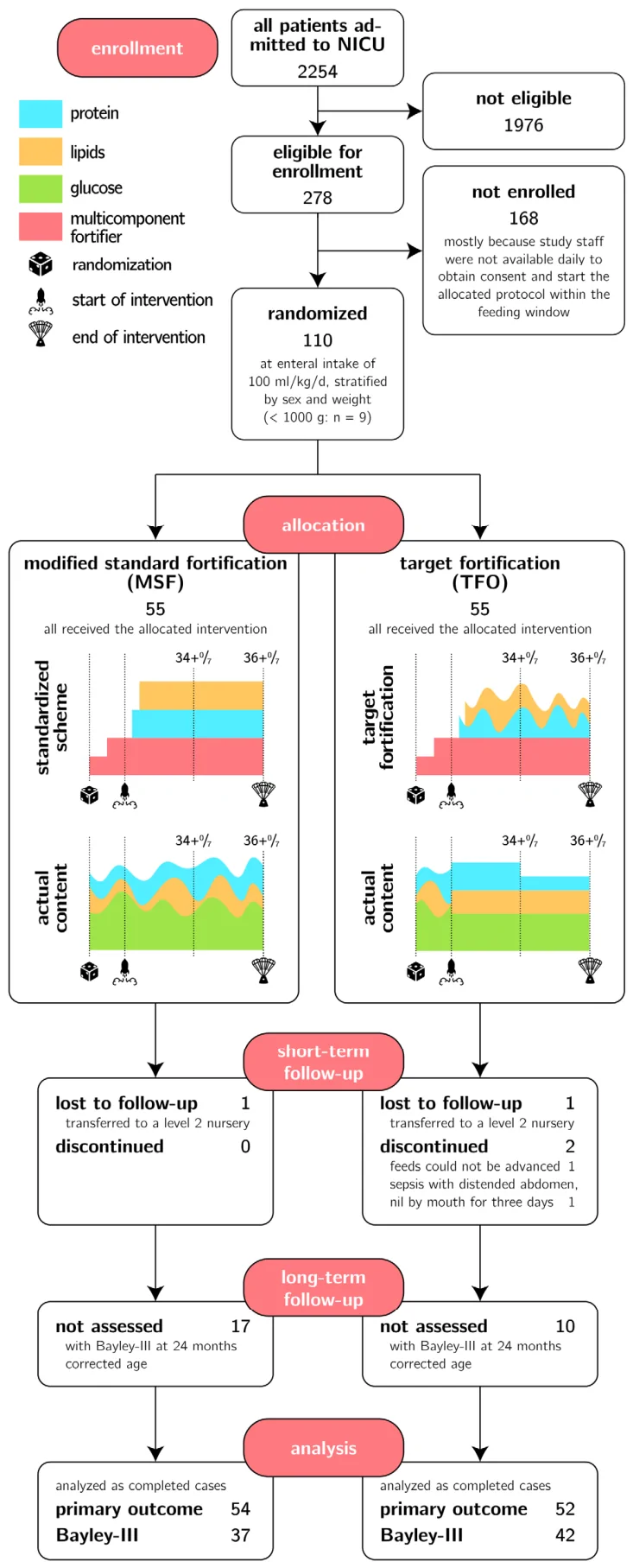
CONSORT flow diagram of admission, eligibility, randomization, allocation, follow-up and analysis, showing the number of eligible infants who were not enrolled, the allocation and the reason for each dropout, the number of infants assessed with the Bayley-III at 24 months corrected age, and the completed cases analyzed.

Of 2254 infants admitted to the NICU during the study period, 278 were eligible and 110 were randomized (Figure 1). The remaining 168 eligible infants were mostly not enrolled because study staff were not available daily, so consent could not always be obtained and the allocated protocol could not always be started within the feeding window.

Of the 110 randomized infants, 55 were allocated to MSF and 55 to TFO. There were four dropouts (Figure 1), one from MSF and three from TFO. Of these, one infant in each group was transferred to a level 2 nursery (study days 3 and 8), and in TFO one infant could not have feeds advanced and one developed sepsis with a distended abdomen and was kept nil by mouth for three days. No infant died. 106 completed cases were available for the final analysis. Still, the sample size exceeded the calculated 36 infants per group.

Baseline characteristics were balanced (Table 1). Only nine infants were still below 1000 g at randomization, seven in MSF and two in TFO (*p* = 0.18). Oxygen therapy was shorter in TFO (20.9 ± 23.6 vs. 32.1 ± 30.3 days, *p* = 0.036) and length of stay tended to be shorter (68.3 ± 17.6 vs. 75.7 ± 23.2 days, *p* = 0.068), while the rate of bronchopulmonary dysplasia did not differ (37% vs. 50%, *p* = 0.23).

### 3.2. Human-Milk Composition and Achieved Intakes

Native human-milk macronutrient content of the daily 24 h human-milk feeding batches did not differ between the groups (Table 2).

Composition was nevertheless highly variable within that overall similarity. For protein the between-infant coefficient of variation in the infant means was 14.1% and the mean within-infant coefficient of variation across days 17.3%; the corresponding values were 13.7% and 14.4% for fat and 1.9% and 2.7% for carbohydrate. Individual pools ranged from 0.4 to 2.6 g protein per 100 mL, from 1.3 to 7.7 g fat per 100 mL and from 4.5 to 8.3 g carbohydrate per 100 mL (Supplementary Table S1). The upper bound on analytical imprecision (Section 2.3) was 15.6% of the mean concentration for protein, 13.9% for fat and 3.0% for carbohydrate (3064, 3091 and 3089 consecutive-day pairs). Applied to the measured composition of each day’s pool, the fixed MSF supplement would have produced a protein concentration more than 0.2 g/100 mL away from the target on 56% of study days (41% before and 75% from 34 + 0 weeks PMA, when the target concentration fell). In a post hoc analysis with the infant as the unit, the day-to-day coefficient of variation in protein intake (around each infant’s own mean before and after 34 + 0 weeks PMA, when the TFO target changed) was 10.6 ± 3.3% in MSF and 7.9 ± 2.4% in TFO (MSF/TFO ratio 1.35, 95% CI 1.20–1.51; *p* < 0.001). The share of days within 4.0–4.5 g/kg/d did not differ (32 ± 18% vs. 36 ± 17%, *p* = 0.22); outside it, intake lay above the range on 52 ± 25% vs. 39 ± 22% of days and below it on 16 ± 16% vs. 25 ± 21%.

Milk taken directly at the breast, and therefore unfortified, accounted for 1.2 ± 2.8% of enteral fluid overall and did not differ between groups (1.1 ± 2.1% in MSF versus 1.4 ± 3.4% in TFO, *p* = 0.62); 32 infants in each group received some milk at the breast.

**Figure 2:**
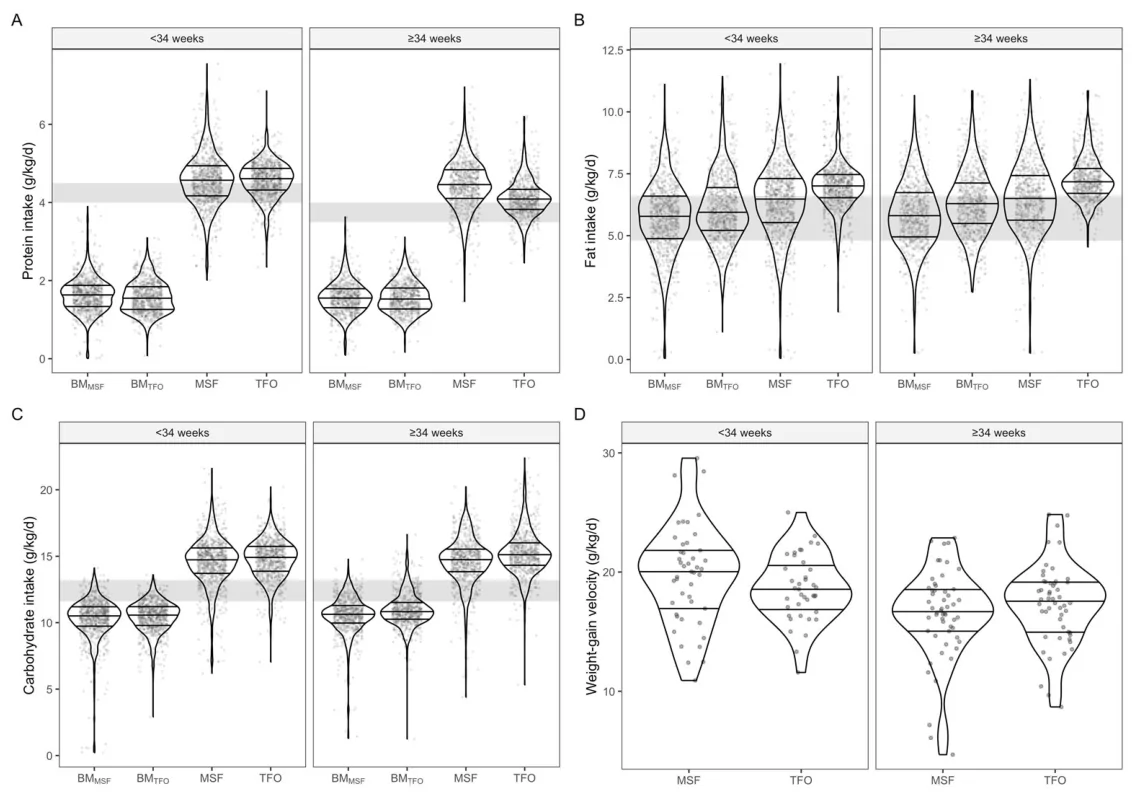
Daily macronutrient intake, expressed per kilogram of body weight and day, from unfortified breast milk (BM) and after fortification (MSF, TFO), below and above 34 weeks PMA: (**A**) protein, (**B**) fat, (**C**) carbohydrate; (**D**) weight-gain velocity. The violins show the distribution of all daily values, with median and quartiles marked and individual daily observations overlaid; the shaded band indicates the target range. Panel (**D**) shows one value per infant and period, computed by the method of Patel and colleagues from the weights at the start and end of that period. Panels (**A**–**C**) show nutrient intake, not the macronutrient concentration of the milk, which is given in Table 2.

Feeding volume was higher in TFO (156 ± 11 vs. 150 ± 19 mL/kg/d, *p* = 0.037). Protein intake was not significantly different before 34 + 0 weeks PMA (4.6 ± 0.4 vs. 4.6 ± 0.3 g/kg/d, *p* = 0.41) but higher in MSF thereafter (4.5 ± 0.4 vs. 4.1 ± 0.4 g/kg/d, *p* < 0.001) (Table 3 and Figure 2); TFO required no protein supplement on 5.4 ± 10.2% of intervention days, and no supplement of any kind on 3.4 ± 8.2%. In MSF the supplement doses actually delivered were 0.98 ± 0.06 g of protein powder and 0.50 ± 0.04 mL of MCT per 100 mL. Computed from the measured native composition and the quantities added, the fortified milk contained 2.96 ± 0.19 g of protein per 100 mL in MSF and 2.78 ± 0.11 g per 100 mL in TFO (*p* < 0.001). TFO received significantly more fat and more energy, while carbohydrate did not differ (Table 3, Figure 2).

**Figure 3:**
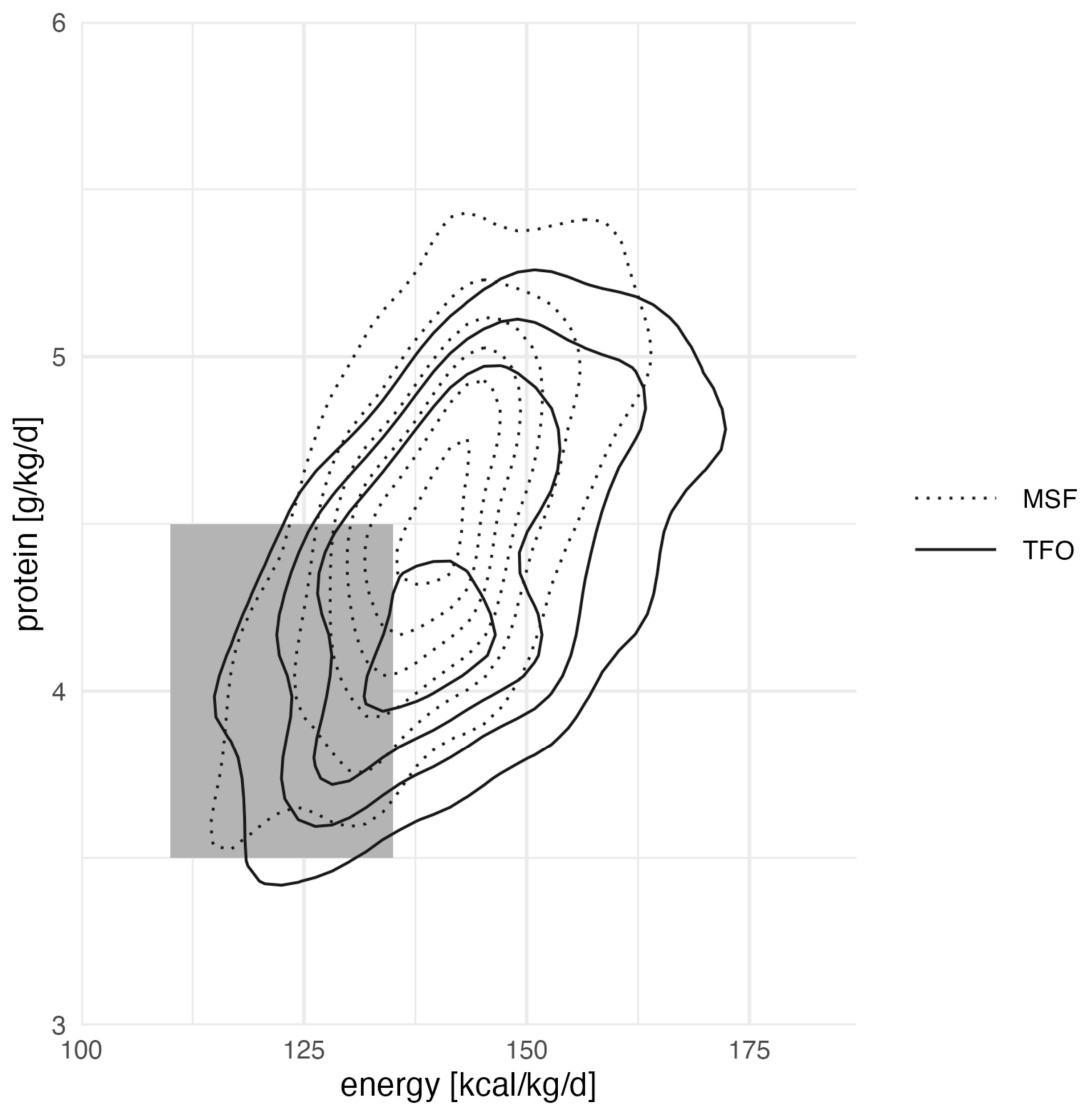
Bivariate distribution of daily protein and energy intake in the MSF (dotted contours) and TFO (solid contours) groups. Contours are two-dimensional kernel density estimates over all daily intake values recorded during the intervention; inner contours enclose regions of higher density. The gray rectangle marks the ESPGHAN 2010 recommendation on which the trial’s targets were based, protein 3.5–4.5 g/kg/d and energy 110–135 kcal/kg/d [17]. The panel shows how protein and energy intake were distributed jointly, not their ratio; group mean intakes are given in Table 3.

The protein-to-energy ratio was higher in MSF (3.2 ± 0.2 vs. 3.0 ± 0.2 g/100 kcal, *p* < 0.001); the two values lie at and just below the 3.2–3.3 g/100 kcal that has been proposed as appropriate for this population [15]. Figure 3 shows the joint distribution of daily protein and energy intake. The gap widened with maturity: before 34 + 0 weeks PMA the ratio was 3.29 ± 0.20 in MSF and 3.22 ± 0.15 in TFO (*p* = 0.05), and from 34 + 0 weeks 3.15 ± 0.43 versus 2.86 ± 0.12 (*p* < 0.001).

### 3.3. Primary Outcome: Growth

Weight-gain velocity did not differ: 17.97 ± 2.27 (MSF) versus 18.14 ± 2.14 g/kg/d (TFO) by the method of Patel and colleagues (MSF − TFO − 0.18 g/kg/d, 95% CI −1.02 to +0.67; *p* = 0.68). Adjusted for sex and weight category at randomization, the difference was −0.21 g/kg/d (95% CI −1.06 to +0.65, *p* = 0.64), and the linear mixed-effects model of the weights measured during the intervention gave a group-by-time difference of +0.17 g/kg/d (95% CI −0.66 to +1.00, *p* = 0.69); both are concordant with the unadjusted comparison.

### 3.4. Secondary Growth, Body Composition and Brain Growth

No differences appeared in weight, length, head circumference, BMI or their z-scores at 36 + 0 weeks PMA (Table 4); weight-, length- and head-circumference-for-age z-scores were −0.69 ± 0.95, −0.86 ± 1.10 and −0.94 ± 0.99 in MSF versus −0.62 ± 0.83, −0.92 ± 1.01 and −0.83 ± 1.02 in TFO. Between-group differences with 95% CI for every outcome in Table 4 are given in Supplementary Table S2. Z-score change during the intervention and individualized growth trajectories gave no diverging contrast, and skinfold thickness and cranial widths did not differ. Nutritive efficiency did not differ either (12.67 ± 3.10 vs. 11.95 ± 1.92 g/100 mL, *p* = 0.15).

### 3.5. Post Hoc Comparison with the Published Protein–Growth Meta-Regression

**Figure 4:**
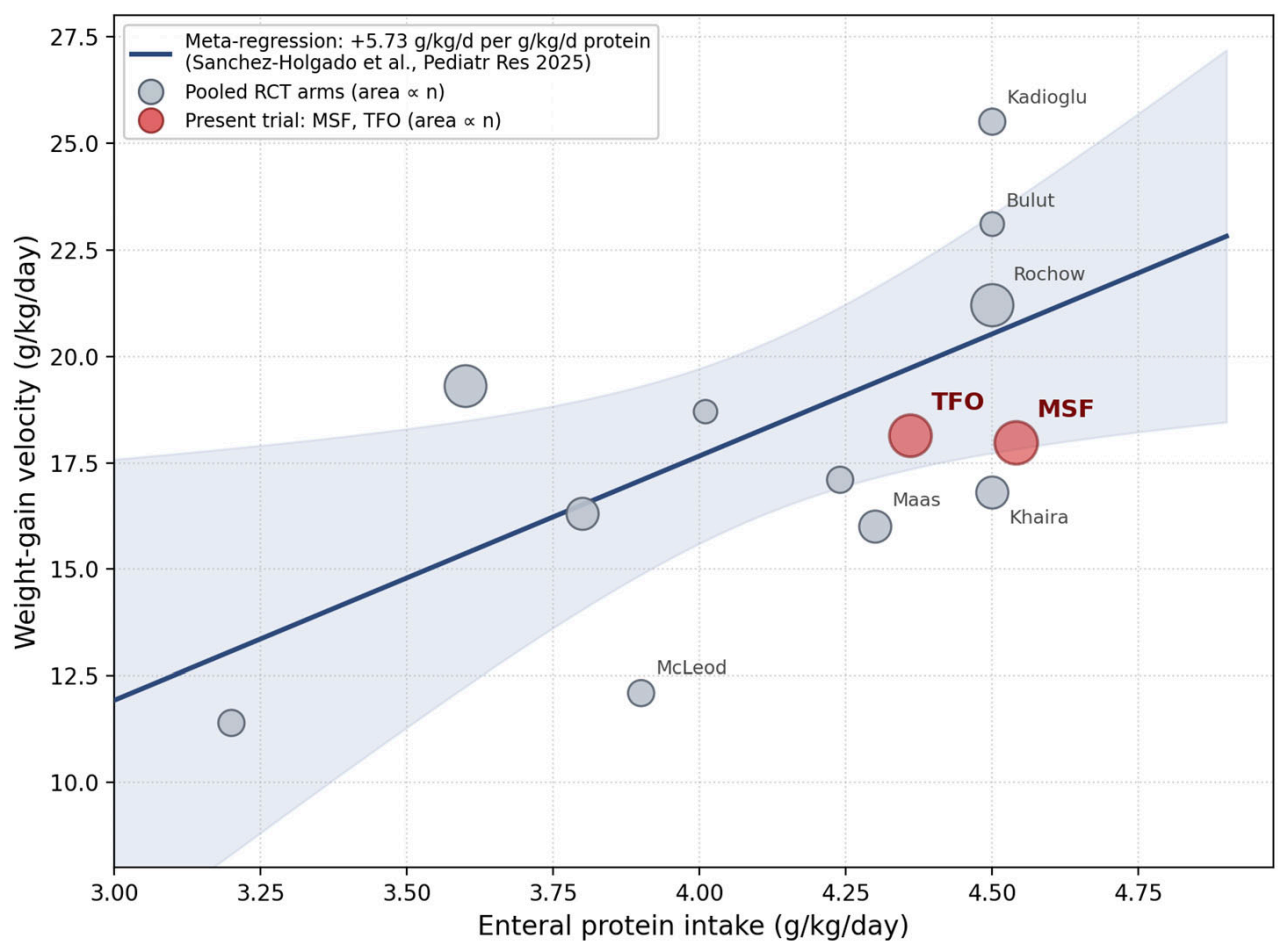
Enteral protein intake versus weight-gain velocity. Gray circles are the individual trial arms pooled in the meta-regression of Sanchez-Holgado and colleagues [12] (slope + 5.73 g/kg/d per g/kg/d protein; area ∝ n), the solid line is the fitted relationship and the shaded area its confidence band. Red circles are the two arms of the present trial; both lie below the fitted line but within its 95% confidence band. This comparison was not prespecified; it is post hoc and hypothesis-generating.

Post hoc, both arms were additionally plotted in the recent weight-gain meta-regression plot of Sanchez-Holgado and colleagues [12]; both lay below the fitted relationship but within its 95% CI band, despite protein intakes at the upper end of the recommended range (Figure 4).

### 3.6. Harms

No infant died, and blood urea, urine calcium, urine phosphorus and urine urea did not differ between groups. The plasma amino acids are reported in the companion paper (Gebauer, Ascherl et al., submitted).

### 3.7. Neurodevelopment

Bayley-III was available for 79 of 106 infants (75%; 37 MSF, 42 TFO); cognitive, language and motor composite scores did not differ (Table 5), and no infant had cerebral palsy or hearing impairment. The 79 infants assessed and the 27 not assessed did not differ in allocation (37 of 54 in MSF versus 42 of 52 in TFO, *p* = 0.22), gestational age at birth (193.9 ± 12.3 versus 195.7 ± 14.1 days, *p* = 0.57), birth weight (0.96 ± 0.26 versus 1.02 ± 0.27 kg, *p* = 0.35) or weight-gain velocity (18.0 ± 2.1 versus 18.2 ± 2.4 g/kg/d, *p* = 0.74), but girls were more likely to be assessed than boys (44 of 52 versus 35 of 54, *p* = 0.03). Attrition was therefore not entirely non-differential, and with a quarter of the cohort unassessed attrition bias cannot be excluded. These analyses are exploratory and under-powered.

## 4. Discussion

### 4.1. Principal Findings

High-protein target fortification did not improve in-hospital growth or 24-month neurodevelopment compared to optimized high-protein standard fortification. Therefore, once a standard regimen has itself been optimized, measuring milk composition daily and adjusting the supplementation added no further benefit with regard to growth. The intra- and interindividual variability that motivates target fortification was present in the milk we fed (Section 3.2), and TFO reduced the day-to-day variability of protein intake by about a quarter, yet this more consistent intake did not result in better growth or neurodevelopment. The gain in precision did not bring more days into the range of 4.0–4.5 g/kg/d, because the level of intake was set by the protein concentration aimed at, not by the precision of fortification: at 150–160 mL/kg/d, 3.0 g/100 mL delivers 4.5–4.8 g/kg/d and 2.6 g/100 mL 3.9–4.2 g/kg/d.

### 4.2. Clinical Relevance of the Null Result

A null result is informative only in relation to the difference that would have mattered. The trial was designed to detect 0.666 × SD, corresponding to about 1.5 g/kg/d given the standard deviation of weight-gain velocity observed here. The difference found was −0.18 g/kg/d (95% CI −1.02 to +0.67 g/kg/d). The 95% CI excludes the difference the trial was designed to detect and clinically relevant differences, while remaining compatible with smaller effects. Over the 34-day mean intervention a difference of 1 g/kg/d corresponds to a few tens of grams of body weight, which we do not regard as clinically important in this population. We therefore read the result as excluding a clinically meaningful growth advantage of target fortification over an intensified standard regimen, rather than as demonstrating equivalence between the two.

### 4.3. Protein–Energy Balance Rather than Protein Alone

The most informative observation is not the null primary outcome but the dissociation between protein intake and growth. MSF delivered more protein (4.5 vs. 4.4 g/kg/d overall, and 4.5 vs. 4.1 g/kg/d after 34 + 0 weeks PMA) and carried the higher protein-to-energy ratio, while TFO delivered more fat and more energy; the arms therefore differed less in the adequacy of any single macronutrient than in the balance between them, but growth was similar. A protein-to-energy ratio of about 3.2–3.3 g/100 kcal, within the ESPGHAN recommendation, has long been proposed as appropriate for VLBW infants [15], and both arms of our study slightly exceeded the corresponding energy-to-protein ratio of 30 kcal/g of protein. In addition, in both groups energy and carbohydrate intake was at or above the upper threshold of the ESPGHAN 2022 recommendations [4]. Most achieved daily intakes lay outside the ESPGHAN 2010 target region for protein and energy on which the trial’s targets were based [17] (78% of days in MSF and 76% in TFO), and neither arm held the protein-to-energy ratio within a narrow band (Figure 3). This is consistent with the ratio mattering more than the amount of protein at intakes in this range, but the trial was not designed to test that hypothesis: the ratio was a consequence of the allocated regimens rather than the randomized variable, and the comparison between the two ratios is therefore observational.

The achievable balance is largely dictated by the products. The 4.4% multicomponent fortifier provided 1.1 g protein together with 2.74 g carbohydrate per 100 mL, so protein cannot be raised without raising carbohydrate energy, and both groups were consequently superfortified with carbohydrate, albeit within the dosing recommended by the manufacturers. That carbohydrate is not simply wasted: energy given as carbohydrate spares protein oxidation more effectively than energy given as fat in enterally fed low-birth-weight infants [30]. It does, however, fix the protein-to-energy ratio in a way the clinician cannot adjust, and Rochow and colleagues proposed that an ideal fortifier would instead add 1.8 g protein, 1.8 g carbohydrate and 0.6 g fat per 100 mL [9]. The amino-acid pattern of bovine-based fortifiers differs from that of human milk as well [31]. Escalating feeding volume when weight stagnates raises all macronutrients and energy together and therefore amplifies an existing imbalance rather than correcting it.

The fat supply needs similar considerations. In addition, the quality of the fat differs as well as its amount. Part of the human-milk batches required additional fat to meet the recommendations for preterm infants, and we used medium-chain triglycerides, which preterm infants absorb readily and which enter the mitochondria without the help of carnitine. Human milk, by contrast, supplies a range of long-chain polyunsaturated fatty acids, among them arachidonic acid and docosahexaenoic acid, which promote growth, regulate inflammation and are central to the development of the brain, the retina and the immune system [32,33,34]. No commercial modular lipid fortifier was available on the German market, so neonatal units often use MCT, lipid formulations intended for intravenous use, or plant oils such as canola oil. Both arms received MCT and the TFO group received more of it, so the additional energy in the TFO arm was preferentially in a form that is rapidly oxidized rather than incorporated into tissue. This may be one reason why the higher energy intake in TFO did not translate into faster weight gain, and it argues for supplementing fat in a form closer to the fatty-acid profile of human milk.

Both arms grew below the published protein–weight-gain meta-regression line, though within its 95% confidence band (Figure 4), at intakes of 4.4–4.5 g/kg/d—the range held necessary for intrauterine growth rates and accompanied by an explicit call to maintain appropriate energy-to-protein ratios [12]—while weight-for-age z-scores fell from −0.28 ± 0.88 and −0.19 ± 0.83 at birth to −0.69 ± 0.95 and −0.62 ± 0.83 at 36 weeks PMA. Protein at the top of the range did not by itself prevent this. That comparison is nevertheless a trial-level one: a meta-regression across study arms describes the relation between arm means and cannot establish what a given intake would do in an individual infant, and its confidence band is wide at these intakes. Figure 4 is presented as a description of where our two arms fall, not as validation of an individual dose–response.

### 4.4. Simultaneous Change in Several Nutrients

The intervention was not a single-nutrient manipulation. Relative to MSF, in the TFO group protein, fat, energy, feeding volume and the protein-to-energy ratio differed, and each of these differences may plausibly affect growth: less protein, more fat, more energy, higher feeding volume, and a lower ratio. The trial therefore cannot isolate the independent effect of individualizing protein, which was its original rationale, and the contrasts may be offsetting. Two consequences follow. First, the null primary outcome is a null for the package as a whole, not for protein adjustment only. Second, the secondary differences—the lower feeding volume in MSF and the higher energy intake in TFO—cannot be attributed to any single component. A trial exactly isolating protein adjustment would have to hold feeding volume, fat and energy equal between arms, which the fortifiers currently available do not permit and which would itself depart from the clinical practice such a trial is meant to inform.

### 4.5. Comparison with Other Trials

Our nutritive efficiency (12.67 g/100 mL in MSF and 11.95 in TFO) was close to the 12.5 g/100 mL of the standard-fortification group of Rochow and colleagues and below the 13.9 g/100 mL of their target-fortification group [9]. In that trial, however, target fortification raised the protein supply above that of standard fortification, whereas both groups of the present trial received a high-protein supply. Unlike others [9,35,36], we found no association between protein intake and plasma or urinary urea, so urea performed poorly as a marker of protein tolerance here [36]. The wider literature points in the same direction as our result: compared with unfortified human milk, multi-nutrient fortification yields only modest in-hospital growth benefit with no demonstrated effect on later outcome [1,11]; systematic reviews of higher protein intake report improvements in weight or length z-scores that are small and inconsistent [3,12,13], and the case for intakes above about 3.5–4.0 g/kg/d remains unsettled [14]; and in a randomized trial additional early amino acids did not improve survival free of neurodisability at two years [37]. Our Bayley-III composite scores lay close to the test norm of 100 in both arms, but with a quarter of the cohort unassessed we do not compare them with those of other cohorts.

### 4.6. Strengths and Limitations

Strengths are daily macronutrient measurement in both arms with daily adjustment in the target arm, a human-milk diet without any formula until 36 weeks PMA, blinded allocation with fortification carried out by separate milk-bank staff, and complete ascertainment of the primary outcome in every infant who remained in the unit. Because the feeds were pooled multi-donor and mother’s own milk, daily rather than intermittent analysis was performed to characterize what was actually fed.

Several limitations qualify the interpretation. Because 54 and 52 infants were analyzed instead of the 36 per group on which the sample-size calculation was based, power to detect 0.666 × SD was 0.92 rather than the 0.80 planned, so the four drop-outs did not compromise the trial. The trial was conducted in a single center and recruited between 2013 and 2016; fortifiers, modular products and feeding practice have changed since, and the human-milk-derived fortifiers and lipid modules now available were not, so the protein-to-energy ratio achievable today may differ from the one studied here. The comparator was a modified, intensified standard regimen rather than routine standard fortification. The population was defined by feeding tolerance: randomization occurred only once enteral feeds had reached 100 mL/kg/d, at a mean postnatal age of 26–28 days and about 31 weeks PMA, so infants who did not tolerate advancing feeds, who were transferred early or who died before that point are not represented, and the findings do not apply to extremely preterm or clinically unstable infants in the early postnatal period, when much of the nutritional deficit accrues. German centers of that period divided around a median of 12.5 days to full enteral feeds [38]; in our own unit the median time to full enteral feeds is now about 10 days (manuscript in preparation). In this cohort 100 mL/kg/d was reached at 27.2 ± 8.5 days (median 25, interquartile range 21–32) and full enteral feeds at 30.7 ± 10.6 days of life (median 29, interquartile range 23–36). The intervention was not late by the standards of its era, although the same protocol applied today would begin earlier. The infants who were eligible but not randomized were largely not enrolled because study staff were not available daily, a logistical constraint unrelated to the infants themselves, so we do not regard the randomized cohort as selected beyond this requirement of feeding tolerance. The nutritional contrast between the arms was correspondingly modest, and several nutrients changed together (Section 4.4).

Weight was recorded at a median interval of seven days rather than the shorter interval originally described; however, the interval did not differ between the groups (MSF 6.7 ± 0.7 vs. TFO 6.7 ± 0.6 days; difference 0, 95% CI −0.2 to 0.3; *p* = 0.752) and therefore cannot bias the comparison. Energy intake includes parenteral carbohydrate and amino acids but not parenteral lipid, which was not recorded separately, so energy is underestimated and the protein-to-energy ratio correspondingly overestimated on days with substantial parenteral nutrition. Parenteral nutrition was given on 215 of 3339 intervention days (6.4%) in 35 infants, similarly in both groups (6.7 ± 16.2% vs. 4.3 ± 9.4% of an infant’s days, *p* = 0.35). Nutritive efficiency relates weight gain to enteral volume alone and is therefore not interpretable for infants whose feeds were withheld for part of the intervention (108 infant-days in 25 infants, 11 in MSF and 14 in TFO); it did not differ between the groups under any summary of daily volume we examined. The proportion of donor milk was not recorded per feed and cannot be reported by group; because every pool fed was analyzed and its measured composition entered the intake calculation, any imbalance in that proportion is contained in the intakes reported. Enteral volume was not capped, which widened the fluid and energy differences between arms. Body composition was assessed only by biceps skinfold thickness and BMI z-score; neither air displacement plethysmography nor dual-energy X-ray absorptiometry was available, so the partition between fat and lean mass—the outcome most directly relevant to a protein-to-energy argument, and the one associated with later neurodevelopment [39]—could not be examined. Neurodevelopmental follow-up was incomplete at 75% and girls were assessed more often than boys, so those analyses are exploratory, under-powered and subject to a sex-related attrition bias. Secondary and harm comparisons were numerous and unadjusted for multiplicity, so individual *p*-values close to 0.05 should not be read as findings, and the meta-regression comparison is post hoc. Nutritional intake was measured daily but entered the analysis as a per-infant summary, without adjustment for the correlation between repeated measurements within an infant. Finally, the TFO group received oxygen for fewer days and left hospital earlier; because chronic pulmonary insufficiency raises energy expenditure and depresses growth [40], less respiratory morbidity would be expected to favor growth in that arm, so this unadjusted post-randomization imbalance cannot explain the absence of a growth difference, although it may have narrowed it.

### 4.7. Implications for Practice and Research

Postnatal growth in VLBW infants is unlikely to be captured by a single macronutrient target. Once a standard regimen has been intensified to guideline intakes, the additional analytical and staffing effort of daily milk analysis did not buy further growth in this population, which is relevant to units weighing the cost of routine milk analysis. Future trials should address ratios as well as amounts, and different targets across postmenstrual age and weight-for-age, should start earlier than the point of established feed tolerance if the early nutritional deficit is to be addressed, and should measure body composition directly rather than inferring it from weight and skinfold thickness. A fortifier permitting protein to be raised without an obligatory carbohydrate load, and a modular lipid supplying long-chain polyunsaturated fatty acids rather than energy alone, would make a stable protein-to-energy ratio achievable in practice.

## 5. Conclusions

Compared with an optimized high-protein standard regimen, high-protein target fortification did not improve growth at 36 weeks postmenstrual age or neurodevelopment at 24 months corrected age. Intensifying protein toward the guideline maximum conferred no measurable growth advantage. These findings do not address individualized fortification against routine standard fortification; they suggest that fortification strategies, and the products they rely on, should be judged by the balance of macronutrients and energy they deliver rather than by protein alone.

## Figures and Tables

**Table 1:** Demographic and baseline characteristics, comorbidities and intervention timeline.

	MSF (*n* = 54)	TFO (*n* = 52)	*p*
Male, *n* (%)	28 (52)	26 (50)	1.000
Small for gestational age, *n* (%)	8 (15)	6 (12)	0.833
Gestational age at birth (weeks)	27.6 ± 1.9	27.9 ± 1.7	0.385
Multiple birth, *n* (%)	9 (17)	14 (27)	0.296
Antenatal steroids, *n* (%)	45 (83)	45 (87)	0.850
Cesarean section, *n* (%)	43 (80)	45 (87)	0.491
Birth weight (g)	960 ± 290	991 ± 238	0.544
Birth length (cm)	34.9 ± 3.5	35.1 ± 2.8	0.697
Birth head circumference (cm)	25.3 ± 2.5	25.1 ± 1.7	0.709
Birth weight z-score	−0.28 ± 0.88	−0.19 ± 0.83	0.600
Weight stratum < 1000 g at randomization, *n* (%)	7 (13)	2 (4)	0.182
Age at study start (days)	28.1 ± 8.5	26.3 ± 8.4	0.279
Duration of intervention (days)	34.7 ± 12.0	33.6 ± 12.4	0.658
Length of stay (days)	75.7 ± 23.2	68.3 ± 17.6	0.068
Duration of oxygen (days)	32.1 ± 30.3	20.9 ± 23.6	0.036
BPD (oxygen at day 28), *n* (%)	27 (50)	19 (37)	0.229
Death, *n*	0	0	—

Data are mean ± SD unless stated. BPD, bronchopulmonary dysplasia.

**Table 2:** Native human-milk macronutrient composition before fortification (mean of each infant’s daily analyses).

	MSF (*n* = 54)	TFO (*n* = 52)	*p*
Milk analyses per infant, *n*	29.9 ± 10.4	30.5 ± 11.5	0.775
Protein (g/100 mL)	1.07 ± 0.15	1.03 ± 0.14	0.154
Carbohydrate (g/100 mL)	6.94 ± 0.13	6.94 ± 0.14	0.922
Fat (g/100 mL)	3.87 ± 0.56	4.02 ± 0.51	0.155

Data are mean ± SD of each infant’s mean daily value, compared by Welch’s *t* test; the unit of analysis is the infant. One sample is one infant-day on which the milk fed was analyzed, from randomization to 36 + 0 weeks postmenstrual age; where two pools were analyzed on the same day, the composition is the volume-weighted mean of both. Readings outside the plausible range for human milk were excluded (14 protein, 1 carbohydrate).

**Table 3:** Macronutrient, fluid and energy intake after fortification (median of each infant’s daily values).

	MSF (*n* = 54)	TFO (*n* = 52)	*p*
Protein intake, all (g/kg/d)	4.5 ± 0.3	4.4 ± 0.3	0.004
Protein intake, <34 wk PMA (g/kg/d)	4.6 ± 0.4	4.6 ± 0.3	0.41
Protein intake, ≥34 wk PMA (g/kg/d)	4.5 ± 0.4	4.1 ± 0.4	<0.001
Fat intake (g/kg/d)	6.5 ± 1.0	7.2 ± 0.6	<0.001
Carbohydrate intake (g/kg/d)	14.7 ± 1.1	15.1 ± 1.1	0.064
Fluid intake (ml/kg/d)	150 ± 19	156 ± 11	0.037
Energy intake (kcal/kg/d)	139 ± 14	144 ± 12	0.029
Protein-to-energy ratio (g/100 kcal)	3.2 ± 0.2	3.0 ± 0.2	<0.001

Data are mean ± SD of each infant’s median daily intake during the intervention, compared by Welch’s *t* test; the unit of analysis is the infant. Rows by PMA include the infants with intervention days in that period (<34 wk: 52 MSF and 50 TFO; ≥34 wk: 53 and 52). PMA, postmenstrual age.

**Table 4:** Growth outcomes at 36 weeks PMA (or discharge, range 34 – 36 weeks).

	MSF (*n* = 54)	TFO (*n* = 52)	*p*
PMA at discharge (weeks)	38.3 ± 2.0	37.4 ± 1.2	0.006
Weight (g)	2377 ± 399	2402 ± 345	0.724
Length (cm)	44.7 ± 2.7	44.5 ± 2.6	0.709
Head circumference (cm)	31.0 ± 1.4	31.2 ± 1.5	0.601
Weight-for-age z-score	−0.69 ± 0.95	−0.62 ± 0.83	0.684
Length-for-age z-score	−0.86 ± 1.10	−0.92 ± 1.01	0.774
Head-for-age z-score	−0.94 ± 0.99	−0.83 ± 1.02	0.571
Difference from individualized growth trajectory, weight (g)	167.3 ± 197.0	170.7 ± 183.8	0.927
Change in weight z-score during the intervention	0.09 ± 0.39	0.14 ± 0.31	0.516
Weight-gain velocity, Patel (g/kg/d)	17.97 ± 2.27	18.14 ± 2.14	0.678
Nutritive efficiency (g/100 mL)	12.67 ± 3.10	11.95 ± 1.92	0.153
BMI z-score at 36 wk PMA	−0.08 ± 0.58	0.11 ± 0.67	0.127
Skinfold thickness (mm)	5.21 ± 1.32	5.12 ± 1.23	0.717
Interhemispheric width (mm)	2.7 ± 1.4	2.5 ± 1.1	0.565
Craniocortical width (mm)	3.5 ± 1.3	3.0 ± 1.2	0.155

Data are mean ± SD. All rows are based on 54 (MSF) and 52 (TFO) infants except skinfold thickness (52 and 52) and interhemispheric and craniocortical width (32 and 30). Between-group differences with 95% confidence intervals are given in Supplementary Table S2.

**Table 5:** Neurodevelopment at 24 months corrected age (Bayley-III).

	MSF (*n* = 37)	TFO (*n* = 42)	*p*
Cognitive composite	96.6 ± 16.7	92.9 ± 15.1	0.30
Language composite	98.0 ± 20.0	94.6 ± 18.4	0.441
Motor composite	102.0 ± 13.3	100.7 ± 14.1	0.66
Cerebral palsy, *n*	0	0	—
Hearing impairment, *n*	0	0	—

Data are mean ± SD. Follow-up was available for 79 of 106 infants (75%); these analyses are exploratory and under-powered.

## Data Availability

Deidentified individual participant data will not be made available. The parental consent obtained between 2013 and 2016 did not cover the sharing of individual participant data beyond the study team.

## Supplementary Materials

The following supporting information can be downloaded at https://www.mdpi.com/article/10.3390/nu18193241/s1, The CONSORT 2025 checklist [41] is provided as Supplementary Material (Supplementary File S1); the CONSORT flow diagram is shown as Figure 1. Supplementary Table S1 reports the between- and within-infant variability of native human-milk composition, and Supplementary Table S2 the between-group differences with 95% confidence intervals for all secondary outcomes. The trial protocol approved by the ethics committee of the Medical Faculty of the University of Leipzig (reference 082-13) is available from the corresponding author on reasonable request.
