# Supplementary material for: Target Versus Modified-Standard Fortification of Human Milk and Growth of Very Low Birth Weight Infants: A Double-Blind Randomized Trial
Source: Nutrients. 2026 Oct 1;18(19):3241. doi: 10.3390/nu18193241 (PMC13667121; doi:10.3390/nu18193241)
Supplement: Supplementary file 1 [file nutrients-18-03241-s001.zip › nutrients-4507657-SupplementaryFileS1-CONSORT-checklist.pdf]

CONSORT 2025 checklist of information to include when reporting a randomised trial

Supplementary File S1 accompanying: "Target versus Modified-Standard Fortification of Human Milk and Growth of Very Low Birth Weight Infants: A Double-Blind Randomized Trial." Trial registration ISRCTN 13923832.

Adapted from: Hopewell S, Chan A-W, Collins GS, et al. CONSORT 2025 statement: updated guideline for reporting randomised trials. BMJ 2025;389:e081123. doi:10.1136/bmj-2024-081123

**Section/Topic**	**Item**	**Checklist item**	Reported in (section)
**TITLE AND ABSTRACT**			
Title	1a	Identification as a randomised trial	Title — identified as a double-blind randomised trial.
Abstract	1b	Structured summary of the trial design, methods, results, and conclusions	Abstract — structured summary of design, methods, results and conclusions.
**OPEN SCIENCE**			
Trial registration	2	Name of trial registry, identifying number (with URL) and date of registration	Title page; Section 2.1. ISRCTN 13923832, https://doi.org/10.1186/ISRCTN13923832. Submitted to the registry on 29 January 2013, before first enrolment on 21 June 2013; publication of the record was delayed to 1 March 2018 because the registration fee was not settled in time by the administering office.
Protocol and statistical analysis plan	3	Where the trial protocol and statistical analysis plan can be accessed	Back Matter — Supplementary Materials. The trial protocol approved by the ethics committee of the Medical Faculty of the University of Leipzig (ref. 082-13) is available from the corresponding author on reasonable request.
Data sharing	4	Where and how the individual de-identified participant data (including data dictionary), statistical code and any other materials can be accessed	Back Matter — Data Availability Statement. Deidentified individual participant data will not be made available; the parental consent obtained between 2013 and 2016 did not cover the sharing of individual participant data beyond the study team.
Funding	5a	Sources of funding and other support (eg, supply of drugs), and role of funders in the design, conduct, analysis and reporting of the trial	Back Matter — Funding. Restricted grant from Milupa Germany, which covered the human milk analyzer (Miris) and its consumables and

			supplied the multicomponent fortifier and the protein supplement; the funder had no influence on design, conduct, collection, analysis, interpretation, writing or the decision to submit. The Funding statement discloses that the funder manufactures the study products (Aptamil Frauenmilchsupplement FMS®, Aptamil Eiweiß+®).
Conflicts of interest	5b	Financial and other conflicts of interest of the manuscript authors	Back Matter — Conflicts of Interest.
**INTRODUCTION**			
Background and rationale	6	Scientific background and rationale	Section 1, Introduction.
Objectives	7	Specific objectives related to benefits and harms	Section 1 (final paragraph) — aim: the effect of target versus modified standard fortification on weight gain. The harms assessment (clinical chemistry, plasma amino acids) is described in Section 2.4; results are summarised in Section 3.6, and the plasma amino acids are reported in the companion paper.
**METHODS**			
Patient and public involvement	8	Details of patient or public involvement in the design, conduct and reporting of the trial	Section 2.1 — no patients or members of the public were involved in the design, conduct or reporting of this trial.
Trial design	9	Description of trial design including type of trial (eg, parallel group, crossover), allocation ratio, and framework (eg, superiority, equivalence, non-inferiority, exploratory)	Section 2.1 — parallel-group, 1:1 allocation, superiority framework.
Changes to trial protocol	10	Important changes to the trial after it commenced including any outcomes or analyses that were not prespecified, with reason	Section 2.1 — no changes to eligibility criteria, interventions or outcomes were made after the trial commenced.
Trial setting	11	Settings (eg, community, hospital) and locations (eg, countries, sites) where the trial was conducted	Section 2.1 — Division of Neonatology, Leipzig University Medical Center, Germany.
Eligibility criteria	12a	Eligibility criteria for participants	Section 2.1 — inclusion criteria (gestational age 24+0–31+6 weeks, birth

			weight 400–1499 g, human-milk feeding without formula) and exclusion criteria (birth asphyxia, major congenital anomaly, abdominal surgery before randomisation, clinically significant renal dysfunction, cholestasis, participation in another interventional trial).
	12b	If applicable, eligibility criteria for sites and for individuals delivering the interventions (eg, surgeons, physiotherapists)	Not applicable.
Intervention and comparator	13	Intervention and comparator with sufficient details to allow replication. If relevant, where additional materials describing the intervention and comparator (eg, intervention manual) can be accessed	Section 2.3 — products, concentrations, targets and escalation schedule for both arms.
Outcomes	14	Prespecified primary and secondary outcomes, including the specific measurement variable, analysis metric, method of aggregation, and time point for each outcome	Section 2.4 — primary outcome: weight gain from the start of the intervention to 36+0 weeks PMA (g/kg/d; Patel exponential method and average accurate daily weight gain). Secondary outcomes, measurement instruments and time points are listed in the same section.
Harms	15	How harms were defined and assessed (eg, systematically, non-systematically)	Section 2.4 — harms were assessed systematically (routine preprandial clinical chemistry every other week; plasma amino acids at 2, 4 and 7 weeks after randomisation). Results are summarised in Section 3.6 and reported in full in the companion paper.
Sample size	16a	How sample size was determined, including all assumptions supporting the sample size calculation	Section 2.5 — standardized effect: 36 completed cases per group for 80% power at $\alpha = 0.05$ (two-sided) to detect 0.666 × SD, the absolute weight gain and its SD being unknown at the design stage; increased to 55 per group to allow for unequal variances and post-randomisation attrition. At the SD observed, 0.666 × SD corresponds to about 1.5 g/kg/d (Section 4.2).

	16b	Explanation of any interim analyses and stopping guidelines	Section 2.5 — no interim analyses were performed and no stopping guidelines were applied.
Randomisation: sequence generation	17a	Who generated the random allocation sequence and the method used	Section 2.2 — randomization in balanced blocks of four, with the allocation sequence prepared in advance and held in sequentially numbered, opaque envelopes.
	17b	Type of randomisation and details of any restriction (eg, stratification, blocking and block size)	Section 2.2 — stratified by sex and actual weight (<1000 / ≥1000 g); permuted blocks of four.
Randomisation: allocation concealment	18	Mechanism used to implement the random allocation sequence, describing any steps to conceal the sequence until interventions were assigned	Section 2.2 — sequentially numbered, opaque, sealed envelopes.
Randomisation: implementation	19	Whether the personnel who enrolled and those who assigned participants to the interventions had access to the random allocation sequence	Section 2.2 — envelopes were opened by the unit secretary, who was not involved in infant care.
Blinding	20a	Who was blinded after assignment to interventions (eg, participants, care providers, outcome assessors, data analysts)	Sections 2.2, 2.4 — parents and NICU staff blinded; the Bayley-III examiner was blinded.
	20b	If blinded, how blinding was achieved and description of the similarity of interventions	Sections 2.2, 2.3 — milk analysis, fortification and handling were performed by separate milk-bank staff.
Statistical methods	21a	Statistical methods used to compare groups for primary and secondary outcomes, including harms	Section 2.5 — Welch two-sample t-test; chi-square test; 95% CIs by Welch approximation.
	21b	Definition of who is included in each analysis (eg, all randomised participants), and in which group	Sections 2.5, 3.1 — completed-case analysis: 106 of the 110 randomised infants (54 MSF, 52 TFO), analysed in the group to which they were allocated; the four dropouts had no evaluable primary outcome.
	21c	How missing data were handled in the analysis	Section 2.5 — no imputation was performed; participants without evaluable data were excluded and are described in the CONSORT flow diagram (Figure 1).
	21d	Methods for any additional analyses (eg, subgroup and sensitivity analyses), distinguishing prespecified from post hoc	Sections 2.5, 3.2, 3.5 — the stratum-adjusted and mixed-model analyses are identified as sensitivity analyses that were not

			prespecified; the variability analysis of daily protein intake (Section 3.2) and the meta-regression comparison (Figure 4) are identified as post hoc; no adjustment was made for multiple comparisons (Sections 2.5, 4.6).
**RESULTS**			
Participant flow (incl. flow diagram)	22a	For each group, the numbers of participants who were randomly assigned, received intended intervention, and were analysed for the primary outcome	Section 3.1; Figure 1 — 2254 infants admitted during the study period, 278 eligible, 110 randomised (55 MSF, 55 TFO), 106 analysed for the primary outcome (54 MSF, 52 TFO).
	22b	For each group, losses and exclusions after randomisation, together with reasons	Section 3.1; Figure 1 — four dropouts with reasons, by arm: MSF, one infant transferred to a level 2 nursery; TFO, one infant transferred to a level 2 nursery, one in whom feeds could not be advanced, and one with sepsis and a distended abdomen, kept nil by mouth for three days.
Recruitment	23a	Dates defining the periods of recruitment and follow-up for outcomes of benefits and harms	Section 2.1 — enrolment 21 Jun 2013 – 31 Aug 2016; follow-up to 24 months corrected age.
	23b	If relevant, why the trial ended or was stopped	Section 2.1 — enrolment continued until the planned sample size had been reached.
Intervention and comparator delivery	24a	Intervention and comparator as they were actually administered (eg, who delivered the intervention/comparator, how participants adhered, whether they were delivered as intended (fidelity))	Section 3.2; Table 3; Figures 2, 3 — achieved macronutrient, fluid and energy intakes by group and by PMA stratum, including the protein-to-energy ratio.
	24b	Concomitant care received during the trial for each group	Sections 2.3, 4.6; Table 1 — parenteral nutrition was included in the daily macronutrient calculation and was given on 215 of 3339 intervention days (6.4%) in 35 infants; comorbidities and concomitant morbidity are reported in Table 1.
Baseline data	25	A table showing baseline demographic and clinical characteristics for each group	Section 3.1; Table 1.

Numbers analysed, outcomes and estimation	26	For each primary and secondary outcome, by group: number analysed; number with available data at the time point; result for each group and the estimated effect size with its precision (eg 95% CI); for binary outcomes both absolute and relative effect size	Sections 3.3, 3.4, 3.7; Tables 4, 5; Supplementary Table S2 — primary outcome MSF − TFO −0.18 g/kg/d (95% CI −1.02 to +0.67), p = 0.68. Bayley-III available for 79 of 106 infants (37 MSF, 42 TFO).
Harms	27	All harms or unintended events in each group	Section 3.6 — no deaths; blood urea, urine calcium, urine phosphorus and urine urea did not differ between groups. Plasma amino acids are reported in the companion paper.
Ancillary analyses	28	Any other analyses performed, including subgroup and sensitivity analyses, distinguishing pre-specified from post hoc	Section 3.5; Figure 4 — post hoc placement of both trial arms against the published protein–weight-gain meta-regression.
**DISCUSSION**			
Interpretation	29	Interpretation consistent with results, balancing benefits and harms, and considering other relevant evidence	Sections 4.1–4.5 and 4.7; Section 5, Conclusions.
Limitations	30	Trial limitations, addressing sources of potential bias, imprecision, generalisability, and, if relevant, multiplicity of analyses	Section 4.6 — single-centre design, 2013–2016 cohort, four dropouts, adjustable feeding volume widening the between-group fluid and energy differences, under-powered neurodevelopmental follow-up, confounded multi-component comparison, and no adjustment for multiple comparisons.
