# Supplementary material for: Target Versus Modified-Standard Fortification of Human Milk and Growth of Very Low Birth Weight Infants: A Double-Blind Randomized Trial
Source: Nutrients. 2026 Oct 1;18(19):3241. doi: 10.3390/nu18193241 (PMC13667121; doi:10.3390/nu18193241)
Supplement: Supplementary file 1 [file nutrients-18-03241-s001.zip › nutrients-4507657-SupplementaryTableS1.pdf]

Supplementary Table S1. Between- and within-infant variability of native human-milk macronutrient content before fortification (g/100 mL).

Component	Infants, n	Infant-days, n	Mean of infant means	Between-infant SD (CV, %)	Within-infant SD (CV, %)	Range of daily values	MSF	TFO	p
Protein	106	3186	1.05	0.15 (14.1)	0.18 (17.3)	0.4–2.6	1.07 ± 0.15	1.03 ± 0.14	0.154
Fat	106	3200	3.95	0.54 (13.7)	0.55 (14.4)	1.3–7.7	3.87 ± 0.56	4.02 ± 0.51	0.155
Carbohydrate	106	3199	6.94	0.14 (1.9)	0.19 (2.7)	4.5–8.3	6.94 ± 0.13	6.94 ± 0.14	0.922

Native (pre-fortification) composition of the pooled milk fed on each infant-day between randomization and 36+0 weeks postmenstrual age, measured by mid-infrared analysis; when two pools were fed on one day, the volume-weighted mean was used. Readings outside 0.4–3.0 g/100 mL for protein, 1.0–9.0 g/100 mL for fat and 4.0–9.5 g/100 mL for carbohydrate were excluded as implausible (14 protein and 1 carbohydrate readings), which is why the number of infant-days differs between rows. The mean of infant means, the between-infant SD and its coefficient of variation (CV) describe the distribution of the per-infant means across the 106 analysed infants; the within-infant SD and CV are the means of each infant's SD and CV across days. Range is the lowest and highest single daily value. MSF and TFO give mean ± SD of the infant means (modified standard versus target fortification); p from Welch's t test on the infant means, unadjusted.
