# Supplementary material for: Target Versus Modified-Standard Fortification of Human Milk and Growth of Very Low Birth Weight Infants: A Double-Blind Randomized Trial
Source: Nutrients. 2026 Oct 1;18(19):3241. doi: 10.3390/nu18193241 (PMC13667121; doi:10.3390/nu18193241)
Supplement: Supplementary file 1 [file nutrients-18-03241-s001.zip › nutrients-4507657-SupplementaryTableS2.pdf]

**Supplementary Table S2.** Secondary growth outcomes at 36 weeks postmenstrual age, with between-group differences and 95% confidence intervals.

| Outcome | n (MSF/TFO) | MSF | TFO | Difference (MSF − TFO, 95% CI) | p |
|---|---|---|---|---|---|
| PMA at last weight in the intervention (weeks) | 54/52 | 35.99 ± 0.04 | 35.98 ± 0.10 | 0.01 (−0.02 to 0.04) | 0.449 |
| Weight at 36 wk PMA (g) | 54/52 | 2377.39 ± 399.02 | 2402.98 ± 344.56 | −25.58 (−169.04 to 117.87) | 0.724 |
| Length at 36 wk PMA (cm) | 54/52 | 44.68 ± 2.69 | 44.49 ± 2.57 | 0.19 (−0.82 to 1.20) | 0.709 |
| Head circumference at 36 wk PMA (cm) | 54/52 | 31.06 ± 1.44 | 31.21 ± 1.54 | −0.15 (−0.73 to 0.42) | 0.601 |
| Weight-for-age z-score | 54/52 | −0.69 ± 0.95 | −0.62 ± 0.83 | −0.07 (−0.41 to 0.27) | 0.684 |
| Length-for-age z-score | 54/52 | −0.86 ± 1.10 | −0.92 ± 1.01 | 0.06 (−0.35 to 0.47) | 0.774 |
| Head-for-age z-score | 54/52 | −0.94 ± 0.99 | −0.83 ± 1.02 | −0.11 (−0.50 to 0.28) | 0.571 |
| Difference from individualized trajectory (g) | 54/52 | 167.28 ± 197.44 | 170.68 ± 183.77 | −3.40 (−76.84 to 70.04) | 0.927 |
| Change in weight z-score during the intervention | 54/52 | 0.09 ± 0.39 | 0.14 ± 0.31 | −0.04 (−0.18 to 0.09) | 0.516 |
| Weight-gain velocity, Patel (g/kg/d) | 54/52 | 17.97 ± 2.27 | 18.14 ± 2.14 | −0.18 (−1.02 to 0.67) | 0.681 |
| Weight-gain velocity, AADWG (g/kg/d) | 54/52 | 17.82 ± 2.99 | 17.94 ± 2.79 | −0.12 (−1.23 to 0.99) | 0.833 |
| Nutritive efficiency (g/100 mL) | 54/52 | 12.67 ± 3.10 | 11.95 ± 1.92 | 0.72 (−0.27 to 1.71) | 0.153 |
| Mean enteral volume (mL/kg/d) | 54/52 | 144.75 ± 22.41 | 150.77 ± 14.36 | −6.03 (−13.26 to 1.21) | 0.101 |
| BMI at 36 wk PMA (kg/m$^2$) | 54/52 | 11.81 ± 0.85 | 12.09 ± 1.01 | −0.28 (−0.64 to 0.08) | 0.130 |
| BMI z-score at 36 wk PMA | 54/52 | −0.08 ± 0.58 | 0.11 ± 0.67 | −0.19 (−0.43 to 0.05) | 0.127 |
| Skinfold thickness (mm) | 52/52 | 5.2 ± 1.3 | 5.1 ± 1.2 | 0.10 (−0.4 to 0.6) | 0.717 |
| Interhemispheric width (mm) | 32/30 | 2.7 ± 1.4 | 2.5 ± 1.1 | 0.20 (−0.5 to 0.8) | 0.565 |
| Craniocortical width (mm) | 32/30 | 3.5 ± 1.3 | 3.0 ± 1.2 | 0.50 (−0.2 to 1.1) | 0.156 |

*Data are mean ± SD. Differences are MSF − TFO with 95% confidence intervals from Welch's t test; p-values are unadjusted for multiple comparisons and these analyses are secondary. n is the number of infants contributing to each row: cranial-ultrasound measurements were available in 62 of the 106 analysed infants and skinfold thickness in 104. Weight, length and head circumference at 36+0 weeks are interpolated values; z-scores use the 2013 Fenton reference and BMI z-score the Olsen LMS tables. Nutritive efficiency is AADWG divided by the mean daily enteral fluid intake per kilogram. PMA, postmenstrual age; AADWG, average accurate daily weight gain.*
